# Biomimetic Nanotechnology: A Natural Path Forward for Tumor-Selective and Tumor-Specific NIR Activable Photonanomedicines

**DOI:** 10.3390/pharmaceutics13060786

**Published:** 2021-05-25

**Authors:** Sushant Prajapati, Taylor Hinchliffe, Vinay Roy, Nimit Shah, Caroline N. Jones, Girgis Obaid

**Affiliations:** Department of Bioengineering, University of Texas, Dallas, Richardson, TX 75080, USA; Sushant.Prajapati@UTDallas.edu (S.P.); Taylor.Hinchliffe@UTDallas.edu (T.H.); vmr150030@utdallas.edu (V.R.); Nimit.Shah@UTDallas.edu (N.S.); Caroline.Jones@UTDallas.edu (C.N.J.)

**Keywords:** biomimetic, specificity, selectivity, photonanomedicine, homotypic, immune, tumor, nanoconstructs

## Abstract

The emergence of biomimetic nanotechnology has seen an exponential rise over the past decade with applications in regenerative medicine, immunotherapy and drug delivery. In the context of nanomedicines activated by near infrared (NIR) photodynamic processes (photonanomedicines; PNMs), biomimetic nanotechnology is pushing the boundaries of activatable tumor targeted nanoscale drug delivery systems. This review discusses how, by harnessing a unique collective of biological processes critical to targeting of solid tumors, biomimetic PNMs (bPNMs) can impart tumor cell specific and tumor selective photodynamic therapy-based combination regimens. Through molecular immune evasion and self-recognition, bPNMs can confer both tumor selectivity (preferential bulk tumor accumulation) and tumor specificity (discrete molecular affinity for cancer cells), respectively. They do so in a manner that is akin, yet arguably superior, to synthetic molecular-targeted PNMs. A particular emphasis is made on how bPNMs can be engineered to circumvent tumor cell heterogeneity, which is considered the Achilles’ heel of molecular targeted therapeutics. Forward-looking propositions are also presented on how patient tumor heterogeneity can ultimately be recapitulated to fabricate patient-specific, heterogeneity-targeting bPNMs.

## 1. Introduction to Biomimetic Nanotechnology

Photonanomedicines (PNMs) are nanoscale drug delivery systems that are based on photodynamic therapy (PDT), a unique treatment modality that leverages visible or near infrared (NIR) light, photosensitizer (PS) molecules and oftentimes molecular oxygen. PNMs, like conventional nanomedicines, have capitalized on the three-decade surge in attention that nanotechnology has received for improving drug delivery, tumor selectivity and treatment tolerability. While these were the principal goals of synthetic nanomedicines, preclinical and clinical results have been highly variable [1]. Furthermore, irrespective of the type of synthetic nanomaterial used, delivery of nanomedicines to tumors appears to reach a plateau [2], whereby further physiological modulation of tumors is required to augment the accumulation and permeation of nanomedicines. Such approaches for physiological modulation include pharmacological vascular and stromal disrupting agents, radiation therapy, focused ultrasound and PDT [3,4,5,6,7]. Given this major bottleneck in synthetic nanomedicines (including PNMs), biomimetic nanotechnology has emerged as an attractive alternative platform for drug delivery. By capitalizing on the mimicry of various biological processes, biomimetic nanomedicines boast prolonged circulation half-lives, reduced immunological clearance, preferential tumor tissue accumulation and permeation, and cancer cell specific recognition. The biological processes recapitulated by biomimetic nanotechnology provide unparalleled opportunities to augment tumor selective drug delivery, enhance the homogeneity of drug distribution within tumors and provide molecular specificity that is akin to synthetic molecular targeted nanomedicines in a manner that is arguably far superior.

**Figure 1:**
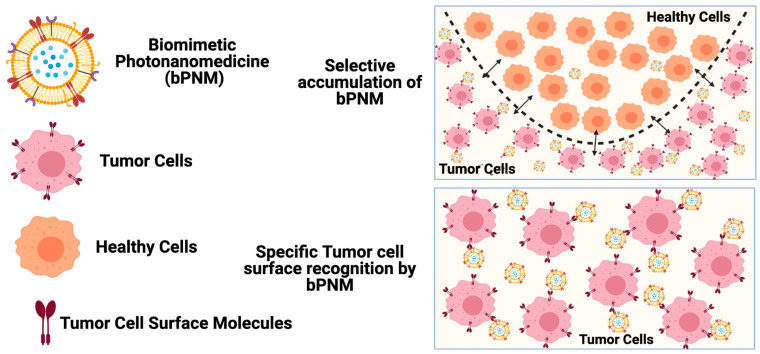
Graphical representation of the differences between tumor selectivity and tumor specificity that is exhibited by biomimetic photonanomedicines (bPNMs) [3].

This review will focus on the unique attributes of biomimetic nanotechnology that allow biomimetic PNMs (bPNMs) to exhibit pronounced tumor selectivity (preferential tumor accumulation) in addition to tumor specificity—discrete molecular affinity for cancer cells through a process known as homotypic recognition [3]. Both tumor selectivity and tumor specificity, being critical for the safety and efficacy of PDT-based combination therapies, hold significant promise as the next generation of nanomedicines in the clinical arena [3]. Figure 1 is a graphical depiction of how bPNMs are capable of exhibiting both tumor specificity and tumor selectivity in solid tumors, which also clarifies the distinction between both concepts. We have previously described in depth how molecular targeted PNMs can exhibit both tumor selectivity (an attribute of nanomedicines) and tumor specificity (an attribute of personalized molecular targeted medicines), and we have conceptually delineated the differences between both concepts [3].

However, while bPNMs are highly attractive, serious considerations must be made on how the use of bPNMs can be actualized in the clinic. The envisaged workflow of how patient tumor cells, or otherwise, can be extracted and used to formulate bPNMs is highly complex and subject to substantial variability and regulatory hurdles. Furthermore, tumor specificity of bPNMs through homotypic recognition has only been demonstrated in pre-clinical studies using cell-line based tumor models. Given the heterogeneity of clinical tumor manifestations, it remains unclear to date if and how patient-derived bPNMs will exhibit molecular specificity through homotypic recognition. Based on current clinical protocols for tumor biopsies, we speculate in Section 4 of this review on how heterogenous patient tumors can be characterized and recapitulated in order to fabricate patient-customized bPNMs that have the potential of tumor-selective and tumor-specific delivery of PDT-based combination regimens.

**Figure 2:**
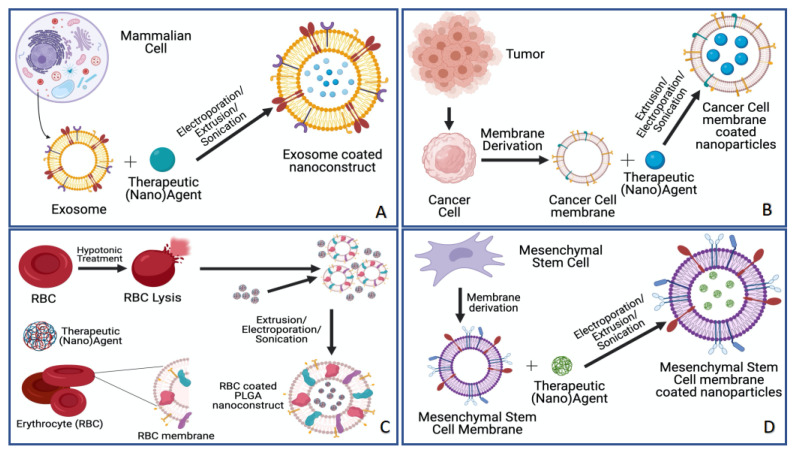
Illustration of different synthetic approaches for biomimetic nanotechnology to improve the tumor selectivity and tumor specificity of therapeutic (nano)agents. These approaches include (**A**) exosome coatings [8,9] (**B**) cancer cell membrane coatings [10,11,12], (**C**) erythrocyte coatings [13,14,15] and (**D**) mesenchymal stem cell (MSC) membrane coatings [16]. These approaches have been used as platforms for bPNMs as discussed in greater detail in Section 2.

In this section, we will summarize key biomimetic nanotechnologies, which are fabricated using various techniques that include the use of exosomes, cancer cells, red blood cells and stem cells (Figure 2). These approaches are of particular significance to this review as they form the basis of several important bPNM studies that are described in detail in Section 2.

### 1.1. Exosomes

Exosomes are 40–100 nm lipid bilayer-enclosed vesicles that can actively cross various biological membranes and barriers, such as plasma membranes and the blood brain barrier [8,17]. They are proactively secreted by a variety of cells (dendritic cells, T cells, epithelial cell, mast cells, etc.) and mediate signal transduction (PI3K/Akt, Wnt signaling, etc.) as well as the exchange of biological and genetic material (SiRNA, proteins, DNA, RNA, etc.) [18,19,20,21]. Unlike other natural drug delivery systems, such as viral capsid carriers, exosomes are weakly immunogenic, non-mutagenic and non-cytotoxic [21,22]. Furthermore, exosomes are superior to synthetic bilayer lipid membrane vesicles, such as liposomes, in that they exhibit longer circulation half-lives, membrane proteins (Lamp2b, CD63 and many others) and enhanced tumor tissue penetration [22,23,24]. More importantly, exosomes exhibit a unique landscape of surface proteins referred to as the surfaceome [25,26,27]. The surfaceome of exosomes dictates their functionality for cell-specific targeting of biological and genetic material. Exosomes can thus be engineered to selectively target tumor tissue through their superior pharmacokinetics, and to specifically target tumor cells at the molecular level through surfaceome-to-cancer cell complementarity.

In recent years, exosomes have emerged as efficient carriers of anti-angiogenic siRNA, spherical nucleic acid (SNA) gold nanoparticle conjugates, doxorubicin-loaded porous silicon nanoparticles, free chemotherapeutics, such as paclitaxel, doxorubicin and curcumin, amongst many other therapeutics and nanotherapeutics [8,17,28,29,30,31]. Furthermore, they can also be surface-modified with additional targeting proteins to increase the specificity of the therapeutic cargo, especially for chemotherapeutics whereby off-target toxicity remains to be a severe impediment. For example, exosomes have been decorated with iRGD to target Integrin αvβ3 on the vasculature of tumors, with LFA-1 for targeting endothelial cell adhesion molecules, and with MHC-II for targeting T-cells, amongst many other ligands [30,32,33]. As such, exosomes are also emerging as a unique bPNM platform that mimics synthetic liposomes but also exhibits an active biological functionality.

### 1.2. Cancer Cell Membrane Coatings

Cancer cell membrane coatings are one of the most prominent biomimetic approaches for oncological applications, which can be readily expanded for large-scale preparation owing to the immortal nature of cancer cells [34]. As with most biomimetic nanotechnologies, cancer cell membrane coatings exhibit prolonged blood circulation times and immune evading characteristics that make them an excellent candidate for drug delivery [34,35]. However, cancer cell membrane coatings possess a unique feature that is commonly referred to as homotypic targeting, whereby the constructs can recognize and bind to the parent cancer cells from which they were derived with molecular specificity [34,35]. Cancer cell membranes can effectively be derived from any cancer cell type; the caveat being only tumors developed from that specific cancer cell type will be targeted by the respective biomimetic construct [10]. Multiple studies have found that cancer cell membrane surfaceomes are equipped with cell–cell adhesion molecules, which facilitate homotypic targeting [35]. The mechanisms underlying homotypic recognition will be discussed in greater detail in Section 3.1. As compared to uncoated nanoparticles, cancer cell membrane coated nanoparticles generally provide greater tumor-selective accumulation, molecular specificity towards cancer cells and reduced accumulation in healthy tissue [34].

Cancer cell membrane coatings have been extensively used for formulating therapeutic drugs, such as doxorubicin and paclitaxel, to treat breast cancer, ovarian cancer, lung cancer and many others [34,36,37]. A variety of other nanoparticles have been delivered with cancer cell membrane coatings, such as indocyanine green-poly(lactic-*co*-glycolic acid), poly(lactic-*co*-glycolic acid) (PLGA), oxaliplatin-containing nanoparticles, tirapazamine-containing nanoparticles, amongst several others [10,35,38,39].

### 1.3. Erythrocyte Membrane Coated Nanoconstructs

Erythrocytes, also known a red blood cells (RBCs), are one of the most abundant cells (on average 5 billion per mL) in the body, providing a rich source for coatings and drug carrier functionalization [40]. Erythrocyte-coated nanoparticles are less susceptible to macrophage engulfment and display prolonged blood circulation [35]. The long circulatory effect is mediated by a series of surface membrane proteins, including CD47, C8 binding protein, CD59 and membrane cofactor protein [40]. RBC membrane carriers have been widely used to encapsulate nucleic acids, proteins and small molecules owing to their intrinsic biocompatible, non-immunogenic and biodegradable nature [41,42].

RBC membrane coatings have been utilized in various cancer therapies, such as paclitaxel and poly(caprolactone) loaded nanoparticles for metastatic breast cancer, glucose oxidase (GOx) and hypoxia-activatable cytotoxic drug tirapazamine (TPZ)-loaded nanoparticles for colon cancer treatment, PLGA for colorectal cancer treatment, PLGA loaded with cycloapmine for pancreatic ductal adenocarcinoma and so on [14,40,43]. Similarly, various other therapeutic materials have been encapsulated for cancer therapy such as free chemotherapeutics and gold nanomaterials, amongst a variety of others [42,44].

### 1.4. Stem Cell Membrane Coated Biomimetic Nanoconstructs

Stem cells are undifferentiated progenitor cells with the capacity to differentiate into specific cell types, depending on their lineages. However, the pluripotency of certain stem cells can be maintained for up to 50-doubling times or more, which enables their engineering for therapeutic purposes. Mesenchymal stem cells (MSCs) and neural stem cells in particular are also known to exhibit tumor-homing properties, whereby they exhibit a natural affinity for the tumor interstitium. It is not entirely clear why MSCs and neural stem cells exhibit these tumor-homing properties, although a gradient of chemokines and chemoattractants produced by cells within the tumor interstitium are known to contribute to the tumor affinity of these stem cells [45,46,47]. While this tumor-homing behavior involves active migratory cellular processes, it has been found that biomimetic stem cell membrane coatings have also been found to exhibit tumor-homing behavior. It is therefore unclear how biomimetic stem cell membrane coatings serve as tumor-selective platforms for drug delivery, although they have in fact proven to be powerful. Stem cell membrane coatings are attractive biomimetic drug delivery systems in that they prolong blood circulation times, are non-immunogenic and have thus been shown to provide the nanoparticles with greater tumor selectivity, along with reduced clearance by the reticuloendothelial system [48,49]. Stem cell membrane coatings have been used to deliver various therapeutic nanoparticles for cancer therapy, such as MSC-coated nanogels loaded with doxorubicin for cervical cancer, MSC-coated PLGA loaded with doxorubicin for liver tumors, MSC-coated PLGA loaded with paclitaxel for orthotopic breast cancer and neural stem cell coated gold nanoparticles, amongst several other free-form and nanoparticulate agents [16,41,45,48,49].

## 2. Biomimetic Nanotechnology in NIR-Activated Photodynamic Therapy

### 2.1. NIR Activable Photonanomedicines

As mentioned in Section 1, NIR activable PNMs are activated by photodynamic processes, which also serve as the foundation for anticancer PDT. While PNMs can contain various PS molecules that are activable by light spanning the UV–visible–NIR spectrum, NIR activable PNMs are particularly attractive due to their tissue-penetrating properties at such wavelengths that lie within the optical windows of biological tissue [3]. Visudyne is the first and only PNM approved by the FDA to date which comprises a lipid-based PNM that contains the NIR activable photosensitizer benzoporphyrin derivative (BPD). Visudyne has laid the foundation for major recent advances in synergistic PDT-based combination therapies. Such synergistic combinations include immunotherapeutic agents [50,51,52,53], chemotherapeutics [54,55,56,57,58,59], small molecule inhibitors [60] and targeted biologics [61,62]. NIR activable PNMs offer a particular advantage in that the PS that is capable of inducing synergistic photodamage to tumor tissue is also responsible for photodynamic tissue priming for secondary therapeutics. Thus, NIR-activated photochemistry can serve as the facilitator for spatiotemporally controlled combination therapies upon light irradiation.

**Figure 3:**
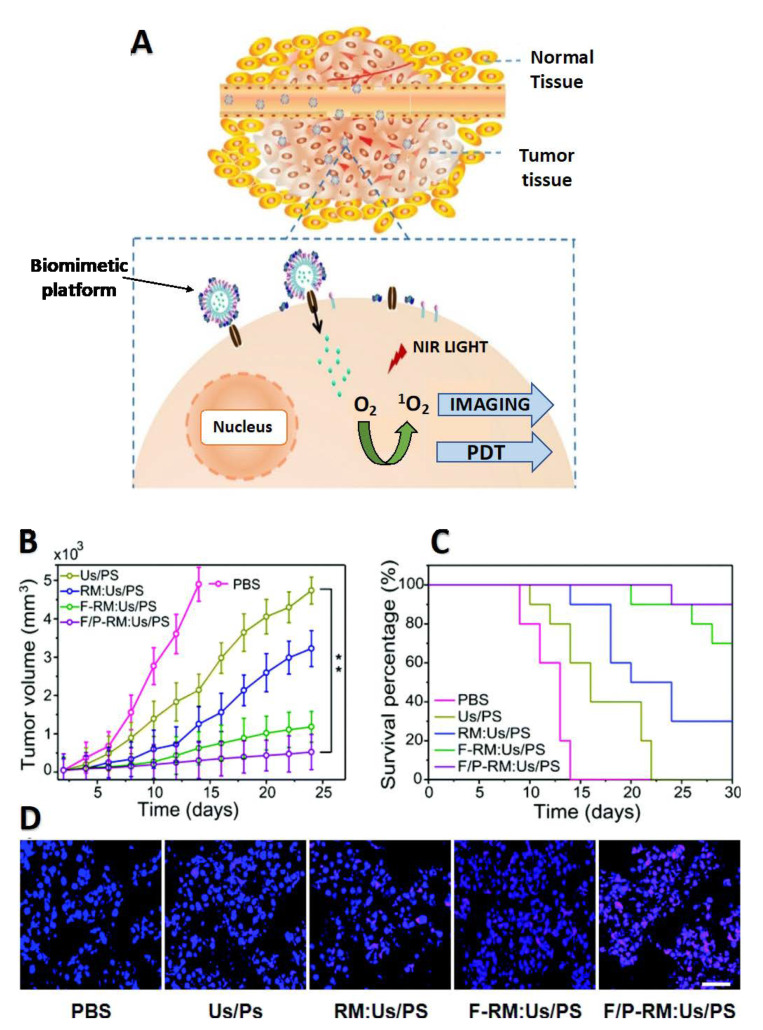
(**A**) Schematic diagram for cell-like biomimetic platform in NIR-activated photodynamic therapy. Adapted with permission from [63], Colloids Surfaces B Biointerfaces 2016. (**B**) Tumor volume of mice for different constructs after PDT treatment. (**C**) Survival percentage of mice in the various treatment groups. (**D**) Cell nuclear morphology for tumor tissue in the respective treatment groups, Scale bar = 50 μm. Adapted with permission from [64], Nanoscale 2020. Acronym definitions: PBS = phosphate buffered saline; Us/PS = upconversion nanoparticles with MC540 photosensitizer molecules; RM:Us/PS = erythrocyte membrane coated Us/PS construct; F-RM:Us/PS = folate decorated RM:Us/PS construct; F/P-RM:Us/PS = RM:Us/PS decorated with folate and mitochondria-targeting triphenylphosphonium.

Various formats of biomimetic nanotechnology are emerging as effective tumor targeting platforms for NIR activable bPNMs. Owing to the amphiphilic and hydrophobic properties of most NIR activable PS molecules used for PDT, they can be readily formulated into the hydrophobic bilayer of bPNMs. Furthermore, biomimetic membranes of bPNMs have also been used to coat nanoparticles entrapping various secondary therapeutic cargos for PDT-based combination therapies. An example of the PDT mechanism of action using bPNMs is depicted in Figure 3A, in addition to how bPNMs can improve tumor photodestruction (Figure 3B), prolong mouse survival (Figure 3C) and enhance cancer cell apoptosis in tumor tissue following NIR activation (Figure 3D). We have previously reported on the powerful attributes of NIR photodynamic activation in the processes that underly the tumor selectivity and tumor specificity of PNMs [3]. In this section, a comprehensive discussion of all NIR activable bPNMs reported to date has been included with special emphasis made on how such approaches have achieved tumor-selective and tumor-specific photodestruction.

### 2.2. Exosomes-Based bPNMs

As described in Section 1.1, Exosomes are promising candidates for biomimetic drug delivery because of their lack of immunogenicity, good biocompatibility, discrete tumor cell interactions and long circulation half-lives, which in turn enhances the drug accumulation at the tumor site [22]. Owing to their small size and ability to cross biological barriers, exosome-based bPNMs can selectively and efficiently deliver therapeutic payloads to tumors, thereby enhancing the PDT efficacy.

To date, there has been only one study using exosome-based bPNMs for NIR activable PDT. This study, by Wang et al., reported bPNMs comprising exosomes derived from mouse MCSs that were loaded with both curcumin (an anticancer agent) and with the NIR PS indocyanine green (ICG). In vivo biodistribution studies in 4T1 tumor-bearing mice 1 h post intravenous administration of the bPNM showed that approximately twice as much curcumin and ICG accumulated in the tumor than when the free agents were administered [65]. At 14 days following NIR irradiation of the tumor (808 nm, 2 W/cm^2^, 10 min), the weight of 4T1 tumors treated with the bPNMs was 4 times lower than with NIR irradiation of the combination of the unformulated free agents. The tumor volume after irradiation was observed to be approximately 8-fold lower with bPNM treatment, as compared to the unformulated and combined free agents. In this study, tumor volumes were calculated as a product of tumor length and the tumor widths squared. In addition, it was also observed that the temperature at the tumor site was raised from 36.1 to 58.6 °C for the mice treated with the bPNM, which was recorded via an infrared thermal imaging camera. This photothermal effect is likely to dominate, and is of course anticipated for a study that uses the common photothermal agent ICG, along with high irradiances, such as the 2 W/cm^2^ used here. As such, the tumor responses here are most likely to be a result of the combined photodynamic and photothermal effects. We have previously discussed the contribution of photothermal effects during a number of NIR-PDT literature reports using common targeted PNMs [3]. While in this study formulation in exosomes did improve the pharmacokinetics and treatment efficacy of the two agents, it remains unclear how tumor-selective and tumor-specific photodestruction by exosome-based bPNMs compares to synthetic PNMs of the same size and drug composition.

### 2.3. Cancer Cell Membrane Coated bPNMs

As mentioned in Section 1.2, cancer cell membrane coatings possessing the unique feature of homotypic targeting of the parent cancer cell, in addition to prolonged blood circulation and immune evasion capabilities. In the context of NIR activable bPNMs prepared using cancer cell membrane coatings, the molecular specificity towards cancer cells is critical for intracellular deposition of cytotoxic reactive molecular species.

Li et al. reported a 4T1 murine mammary cancer cell membrane coated bPNM loaded with the hypoxia-activatable cytotoxic drug Tirapazamine (TPZ) and an NIR activable PS (PCN-224) [66]. The 4T1 bPNM was found to specifically recognize 4T1 cancer cells over irrelevant COS7, HepG2, CT26 cells in vitro. The in vivo studies of 4T1 breast tumor accumulation revealed that the bPNM was twice as efficient at tumor selective delivery than the uncoated cocktail of TPZ and PCN-224. When irradiated with NIR light (660 nm, 220 mW/cm^2^, 10 min), the 4T1 tumors treated with the biomimetic system were most responsive to the PDT-based combination at 21 days following therapy. Final assessment of tumor weights revealed that the bPNM was 6-fold more effective than the uncoated cocktail of free TPZ and PCN-224 following NIR activation. Similarly, the tumor volume was found to be approximately 8-fold lower for the bPNM-treated group as compared to the uncoated combination of TPZ and PCN-224. In this study, tumor volumes were calculated using the following equation: (Volume = Width^2^ × length of the tumor/2).

In a separate study by the same team, it was found that 4T1 membrane bPNMs encapsulating the same PS PCN-224, as well as glucose oxidase and catalase, bound to 4T1 cells 2.8-fold more efficiently than irrelevant COS7 cells [67]. It was also found that the bPNM accumulated twice as efficiently as the unformulated PCN-224 in 4T1 tumors. This greater tumor accumulation resulted in an improved antitumor PDT response for the bPNM (97.1% tumor growth inhibition) than for the free PS (67.5% tumor growth inhibition) when activated with NIR light (660 nm, 150 mW/cm^2^). The bPNM elicited a 4-fold reduction in tumor volume as compared to the free PS under the same NIR irradiation parameters. In this study, tumor volumes were also calculated using the following equation: (Volume = Width^2^ × length of the tumor/2).

In a study by Jiong Li et al., bPNMs were synthesized from SMMC-7221 human hepatocellular carcinoma cell coatings that encapsulated a magnetic nanobead that was loaded with the photosensitizer chlorin e6 (Ce6) [68]. Two hours following intratumoral administration in mice bearing SMMC-7721 tumors, photoirradiation was performed (670 nm, 1 W/cm^2^, 10 min). It was found that the bPNM provided a 4-fold greater reduction in tumor weight than the uncoated Ce6 magnetic nanobead. The bPNM also showed 3-fold reduction in tumor volume as compared to the uncoated Ce6 magnetic nanobeads. As with the study described above by Wang et al., the irradiance of 1 W/cm^2^ used in this study is typically associated with photothermal therapy, which is one to three orders of magnitude higher than irradiances commonly used for PDT. However, the contribution of the photothermal effect in this study cannot be quantified, as no thermometry was performed on irradiated tumors.

In a separate study by Yang et al., SGC7901 gastric cancer cell membranes were used to coat silica nanoparticles containing Ce6 [69]. Upon laser irradiation (680 nm at 1 W/cm^2^ for 5 min) of mice bearing SGC7901 tumors, intravenously administered bPNMs resulted in a 6–8 fold reduction in tumor volume, as compared to free Ce6 and the uncoated constructs. As above, the photothermal effect is also expected to have contributed to the reduction in tumor volumes using the irradiance of 1 W/cm^2^. Pan et al. also used a Ce6-based bPNM entrapping a hollow manganese dioxide nanoparticle with glucose oxidase [70]. The bPNM was coated with B16.F10 mouse melanoma cell membranes and provided 8-fold more potent B16.F10 tumor burden reduction than free Ce6 following photoirradiation (655 nm at 100 mW/cm^2^ for 5 min). Aside from the NIR activable bPNM systems we describe in this section, other cancer cell membrane bPNM systems using shorter wavelengths of visible light have also been shown to augment tumor-selective and tumor-specific photodestruction [71].

While the bPNMs in the studies mentioned here unanimously exhibited either molecular specificity towards the parent cancer cells, selective tumor delivery in vivo, or both, it remains unclear at this point how these bPNM system would compare to a synthetic molecular targeted PNM equivalent. However, it is anticipated that the pharmacokinetic properties of the bPNM would be superior and the molecular specificity arguably more relevant for heterogenous tumors than when using a single target synthetic molecular targeted PNM.

### 2.4. Erythrocyte Membrane Coated bPNMs

Due to the origins of their biomimetic coating, nanoconstructs coated with erythrocyte membranes possess prolonged circulation half-lives and enhanced immune evasion from macrophages, making them unique bPNM platforms.

In a study by Ding et al., upconversion nanoparticles containing the PS MC540 were coated with murine red blood cell membranes [64]. The red blood cell membranes were further decorated with folate for molecular targeting of the folate receptor and with triphenylphosphonium for targeting of organelles. Twenty days following NIR PDT (980 nm, 0.5 W/cm^2^, 3 min), B16 murine melanoma tumor volumes were found to be 10-fold lower with the bPNM treatment than with the uncoated constructs, as measured by digital calipers. Furthermore, tumor volumes were 6-fold lower with the bPNM treatment than when the red blood cell membrane coatings were not decorated with folate and triphenylphosphonium. This bPNM construct therefore facilitated tumor-selective delivery as a result of the red blood cell membrane coatings, in addition to molecular specificity through the folate receptor targeting.

Similarly, in a separate study by Zhao et al., an RBC coated metal-organic framework was designed with ferric oxide and ferric-tetra(4-carboxyphenyl) porphyrin as a PS and an AS1411 aptamer for molecular specificity towards nucleolin [72]. They reported that, upon light irradiation (660 nm laser at a current of 1.5 amperes, 10 min irradiation), the RBC bPNM showed approximately 2-fold and 4-fold reduction in KB cervical tumor volume, as compared to free PSs and RBC coated framework without light irradiation, respectively. The tumor volumes in this study were calculated using the following equation: tumor volume = (tumor length × tumor width^2^)/2. However, only marginal improvements in tumor photodestruction were provided by the RBC membrane coating. Thus, the role of tumor selectivity by RBC coatings and the molecular specificity by nucleolin targeting remain somewhat unclear.

Several other RBC-coated bPNMs have also been reported for NIR activable PDT. These include RBC membranes coating both a paclitaxel (PTX) dimer and the PS 5,10,15,20-tetraphenylchlorin (TPC), RBC membranes coating the hypoxia-activatable cytotoxic drug TPZ, the reactive oxygen species prodrug arylboronic ester, and the PS Ce6, amongst others [73,74].

Although RBC coatings for bPNMs have shown to be efficient in delivering PSs and combination therapies, the extent to which they enhance tumor-selective PDT has been mixed. Furthermore, RBC coatings for bPNMs should in theory prolong circulation half-lives; however, this has not been evaluated in the context of NIR activable PDT. This is of particular importance for PSs, whereby their amphiphilic and hydrophobic nature can readily allow them to escape from the bPNMs prematurely. In such instances, the true advantage of RBC coatings for bPNMs may not be fully actualized. This remains to be an important point of investigation for such bPNM systems and their respective PSs selected.

### 2.5. Stem Cell Membrane Coated bPNMs

Since stem cell coatings are non-immunogenic, provide a long circulation half-life and exhibit natural tumor homing properties, they serve as a unique bPNM platform for tumor targeted delivery of PS molecules and their respective combinations. Human and rat bone marrow derived MSCs were used by Gao et al. to prepare membrane encapsulated mesoporous silica-coated upconversion nanoparticles loaded with the PSs ZnPC and MC540 [75]. The MSC membrane bPNMs exhibited a 4.7-fold increase in uptake in HeLa cervical cancer cells, as compared to uncoated counterparts. This suggests that a specific interaction between MSC membranes and cancer cells may exist, although the molecular basis for that interaction remains elusive. After NIR irradiation (980 nm, 0.35 W/cm^2^, 15 min) of mice bearing HeLa tumors, it was found that the antitumor efficacy of the MSC membrane bPNMs was twice as effective as the uncoated constructs. In a separate study by Fen et al., the anti-tumor efficacy of rat MSC membrane coated bPNMs containing gelatin nanogels with Ce6 as a PS was explored in an A549 lung tumor model [76]. Upon NIR laser irradiation (660 nm, 0.5 W/cm^2^, 5 min), the MSC membrane bPNM was most effective at A549 cell cancer photodestruction in vitro with an IC_50_ value of 0.14 μg mL^−1^ as compared to the uncoated nanogel (0.51 μg mL^−1^). The improved cancer cell phototoxicity is again ascribed to a specific molecular interaction between the MSC membranes and the cancer cells. In vivo biodistribution studies in an A549 lung tumor model revealed that the MSC membrane bPNMs were twice as effective at tumor accumulation than the uncoated PNMs 8 h following intravenous administration. While results of the studies using MSC membrane bPNMs show significant promise, a deeper understanding into why such bPNMs exhibit tumor-homing properties is critical for the success of their future translation.

### 2.6. Biomimicry Using Modified Liposomes

Liposomes, phospholipid bilayered membrane vesicles, are of the oldest nanoplatforms used for drug delivery as a result of their biomimicry of cellular membranes. Attractive attributes include high versatility in size control, surface functionality, payload flexibility and tumor-selective delivery. Visudyne, the first and only PNM approved to date, is based on a liposomal format [3,77]. While liposomes are generally synthetic, their biomimicry of cellular membranes can be enhanced by surface modification using biomolecules. This section will discuss key studies that have leveraged modified liposomes as a tumor-selective bPNM platform.

In a study by Ichikawa et al., the authors presented a liposomal formulation of benzoporphyrin derivative monoacid ring A that is analogous to Visudyne [78]. However, the liposomes were PEGylated and surface-modified with the Ala-Pro-Arg-Pro-Gly (APRPG) pentapeptide, which confers specificity for vascular endothelial growth factor receptor-1 that is overexpressed on tumor endothelial cells. Upon laser irradiation (689 nm, 150 J/cm^2^, 0.25 W) following intravenous injection into Meth-A-sarcoma bearing mice, tumor volume reduction was observed to be approximately 3.5-fold for modified liposome as compared to unmodified PEGylated liposome without the pentapeptide. In this study, the tumor volume was measured using slide calipers for two bisected diameters. The pentapeptide facilitated specific tumor delivery of the bPNM for anti-angiogenic and anti-tumor PDT, whereby the liposomal format assisted with selective tumor accumulation.

In another study by Oku et al., the authors reported a liposome modified with palmityl-d-glucuronide in order to increase its circulation half-life and improve the Meth-A-sarcoma tumor-selective delivery of benzoporphyrin derivative monoacid ring A [79]. Under NIR light irradiation using 690 nm, 180 J/cm^2^, 19 min on Balb/c mice bearing Meth-A-sarcoma, it was reported that there was around 1.6-fold and 3.3-fold enhancement in tumor volume reduction using the bPNM, as compared to unmodified liposomes and free PS, respectively. The tumor volume in this study was monitored by slide calipers. Tumor selectivity here was augmented by promoting the bPNM circulation half-life through surface modification.

Liu et al. also reported a biomimetic platform formed by the hybridization of murine macrophage cell membrane with liposomal membranes that were loaded with nano-platinum and the PS benzoporphyrin derivative [80]. A 2.7-fold enhanced accumulation of the hybridized liposome within the 4T1 tumor parenchyma was observed, as compared to the unhybridized liposomes. Upon laser irradiation (690 nm, 100 W/cm^2^, 10 min), it was reported that there was approximately a 2-fold reduction in tumor volume by the hybridized liposomes, as compared to the unhybridized liposomes in intravenously injected orthotopic 4T1 tumor-bearing mice. Tumor volume (V) was assessed using the formula: V = (length × tumor width^2^)/2. While it is clear that the murine macrophage cell membrane hybridization effectively enhanced tumor selective delivery, it is unclear if molecular interactions between the macrophage membrane hybridized liposomes and the cancer cell surface facilitated any tumor-specific delivery and PDT efficacy.

## 3. General Mechanisms of Tumor Specific and Tumor Selective Biomimetic Delivery

### 3.1. Conferring Tumor Specificity through Homotypic Recognition

**Figure 4:**
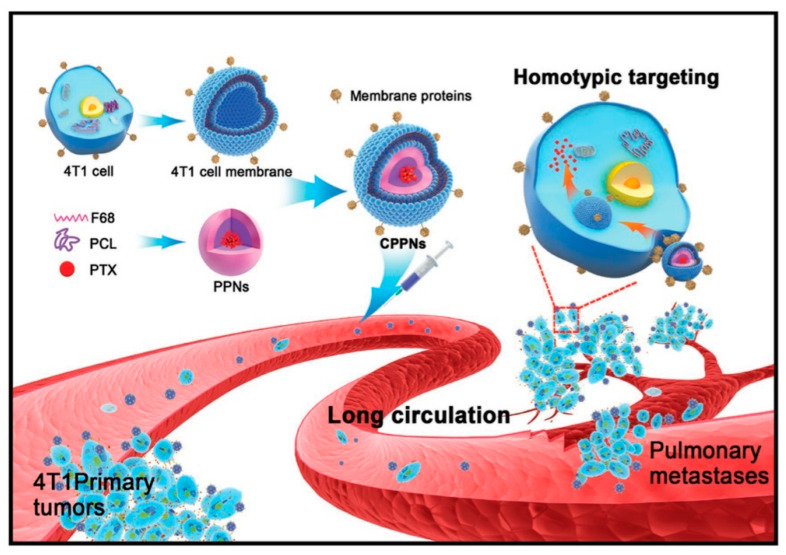
Schematic representation of cancer cell membrane-coated biomimetic nanoparticles for homotypic targeting of 4T1 cancer cells in primary as well as metastasized tumors. Reprinted with permission from [12], Advanced Materials, 2016.

Given the ambiguity of the mechanisms through which cancer cell derived biomimetic constructs, such as exosomes and membrane coated constructs, can recognize and bind to their parent cancer cells, much is left to be explored with regard to the mechanisms of homotypic targeting. The significance of homotypic targeting is further accentuated by the ability of such cancer cell derived biomimetic constructs to recognize and bind to both primary and metastatic tumors (Figure 4). The molecular specificity provided by homotypic targeting is especially critical for the effective photodestruction by bPNMs, as without cancer cell binding and internalization, PDT efficacy is significantly diminished [3,81]. It is conceivable that cancer cell derived biomimetic constructs bind to their parent cancer cells in vitro and in vivo through cell–cell adhesion molecules. These molecules constitute what is known as the surfaceome; the molecular landscape of cancer cell surfaces. However, while these cell–cell adhesion molecules that exist in the surfaceome of cancer cell derived biomimetic constructs are not unique to a specific cell type, let alone cancer cell type, homotypic recognition is exclusively for the parent cancer cells, and not for irrelevant cancer cells. As such, we hypothesize that a steric complementarity must also exist between the cancer cell derived biomimetic constructs and the parent cancer cells from which they were derived. Such steric complementarity between cancer cell derived biomimetic constructs and parent cancer cells likely involves (1) the expression levels, (2) the relative surface densities, and (3) the respective molecular diversity of cell–cell adhesion molecules. However, this steric complementarity has not been explored to date and will likely prove to be pivotal for the successful translation of biomimetic nanotechnology. This section explores the most recent findings on the mechanisms of homotypic tumor recognition which facilitates the unique tumor specificity (cancer cell molecular recognition) that such biomimetic constructs exhibit.

In order to better understand the mechanisms of homotypic targeting of biomimetic constructs, both the presence and abundance of specific cell adhesion molecules must be well understood. Furthermore, the steric complementarity is of paramount importance to identify the parameters necessary for biomimetic constructs to specifically bind to parent tumor cells. While an array of cell adhesion molecules is expressed on the surface of cancer cells, to date, only a certain number of these have been implicated in homotypic targeting. The Thomsen–Friedenreich (TF) antigen, E-Cadherin, N-Cadherin, EpCAM, Na^+^/K^+^-ATPase and glycoprotein 100 (gp100) have all been implicated in homotypic recognition. These molecules all naturally facilitate cell–cell adhesion of diverse cells, including cancer cells, both to each other and to other supportive cells. Their inherent role in mediating the structural integrity of tumors, and in invasion, metastasis and extravasation, might explain how cancer cell derived biomimetic nanoconstructs exhibit specific binding to parent cancer cell lines, in addition to immune evasion and extravasation through intact vasculature.

In a study that analyzed 4T1 membrane-derived biomimetic constructs, TF-antigen, E-cadherin and CD47 were found to be conserved from the parent 4T1 cells. This was confirmed through both sodium dodecyl sulfate polyacrylamide gel electrophoresis (PAGE) and Western blotting [12]. Approximately 90% of human cancer types and the majority of precancerous legions express the TF antigen, a truncated *O*-linked cell surface glycan that is only found on developmental cells and cancer cells [82]. The specific interaction between the TF antigen and surface galactin-3 facilitates cancer cell–cell adhesion, to vascular endothelial cells and to the ECM (fibronectin, collagen, and laminin, etc.) [83]. Cadherins are calcium-dependent cell adhesion proteins and possess a large extracellular domain which binds to the same cadherin molecules present on an adjacent cell. The adhesion of E-cadherins specifically is initiated by the dimerization of two cadherin molecules that results in stable and strong adhesive forces [84]. CD47, as we discuss in greater depth in Section 3, is a self-marker that is primarily involved in immune evasion of biomimetic constructs but not in homotypic recognition.

In another study by Jia et al., ICG encapsulating biomimetic constructs embedded with a heterogenous mixture of C6 glioma cell membrane proteins were capable of successful homotypic targeting of C6 tumors in vivo following active penetration through an intact blood brain barrier [85]. Homotypic targeting in vitro resulted in 3.9 to 7.9 times greater binding of the biomimetic constructs for C6 cells than for other irrelevant, non-target heterotypic cancer and non-cancer cells. These heterotypic cells included HepG2 (human hepatocellular carcinoma cells), MCF-7 (human breast cancer cell), B16 (murine melanoma cells), U87 cells (human glioma cells) and bEnd.3 (murine normal brain microvascular endothelial cells). Furthermore, orthotopic C6 tumor selectivity was found to be 8.4-fold 7 days post implantation, while the BBB was still intact. Synthetic liposome controls did not accumulate in tumors at the same timepoint. While this study demonstrated that cancer cell membrane proteins were responsible for homotypic recognition and facilitated an improvement in phototherapeutic efficacy, it did not identify the specific membrane proteins involved. The findings, however, do suggest that the molecular and steric complementarity of the C6 cell membrane proteins also involves interactions between the construct and angiogenic tumor endothelial cells, which can explain the BBB-permeating characteristics they possess.

In a study by Sun et al., paclitaxel-loaded polymeric nanoparticles coated with 4T1 breast cancer cell membranes exhibited homotypic recognition of primary and metastatic 4T1 tumors in vivo [12]. The authors determined that the basis for homotypic recognition included the cell surface expression of the TF-antigen and E-cadherin, which were present on both the 4T biomimetic construct and the 4T1 parent cancer cells from which they were derived. In a study by Zhu et al., it was found that the surface proteins responsible for the specific homotypic targeting of homologous cancer cells were cadherins, Na^+^/K^+^ ATPase and glycoprotein 100 (gp100) [86]. Hazan et al. also reported that the cadherins were integral to homotypic targeting [84]. In addition to pumping Na^+^ and K^+^ across cell membranes, Na^+^, K^+^-ATPase has also been proposed to contribute to the stability of cell–cell adhesion at adherins junctions [87]. Since the β1 subunits of Na^+^, K^+^-ATPase are thought to form bridges across these junctions, this intercellular adhesive behavior may contribute to homotypic recognition. Glycoprotein 100 (gp100), also known as Pmel17, is an integral membrane glycoprotein synthesized in melanocytes [88,89]. Since it has also been found to accumulate on intralumenal vesicles that can be released as exosomes in cases of melanoma, and melanoma exosome release has been attributed to preparation of the tumor microenvironment for metastasis, gp100 may facilitate homotypic recognition by contributing to intercellular signaling in cancer [88,89].

In another study on biomimetic constructs by Chen et al., MCF-7 cell membrane coated nanoparticles loaded with ICG/poly (lactic-*co*-galactic acid) (PLGA) were tested for homologous targeting [10]. In tumors, it was reported that the cancer cells express certain surface adhesion molecules such as N-cadherin, galectin-3 and epithelial cell adhesion molecule (EpCAM), which are responsible for multicellular aggregate formation. Here, the molecules responsible for the cellular adhesion and specific tumor recognition were reported to be EpCAM, N-cadherin and galectin-3, which were present on the cancer cell surface. Western blotting was conducted to confirm the retention of the homotypic adhesion molecules on the biomimetic construct. The same proteins EpCAM, N-cadherin and galectin-3 were also reported in a study by Liu et al., where biodegradable cancer cell membrane-coated mesoporous copper/manganese silicate nanospheres (mCMSNs) retained the same surface molecular expression patterns as the MCF-7 parent cells [90]. Similar to Na^+^, K^+^-ATPase, epithelial cell adhesion molecule (EpCAM) is also typically found at intercellular junctions and affects cell adhesion as well as a variety of other processes [91]. Since it is upregulated in some cancers and contributes to metastatic behaviors such as proliferation and migration, it may likewise help to confer homotypic recognition between biomimetic carriers and cancers with high EpCAM expression levels.

There have been significant strides in defining the molecules within the surfaceome that contribute to homotypic recognition. However, much is to be left to be explored with regards to the fundamental mechanisms of steric complementarity that we hypothesize is in fact central to the unique homotypic recognition process.

### 3.2. Augmenting Tumor-Selective Delivery through Immune Evasion

Without considering the barriers of nanomedicine extravasation into tumors, it is well established that the efficiency of nanomedicine tumor delivery is directly related to their circulation half-lives. Furthermore, it is well established that their circulation half-lives are directly impacted by the capture of circulating nanomedicines by circulating macrophages, resident macrophages in the liver and spleen and tumor-associated macrophages. As such, immune evasion becomes a critical attribute of biomimetic coatings that offers bPNMs the prolonged circulation half-lives they need for efficient tumor delivery. Studies thus far unanimously demonstrate that biomimetic coatings are superior to the long-hailed stealth PEG coatings that are used for the vast majority of nanomedicines. Owing to the enhanced permeability and retention effect, as well as efficient tumor endothelial transcytosis, both which are unique to tumor tissue, prolonging the circulation half-lives of by biomimetic coatings will inadvertently improve bulk tumor delivery and tumor selectivity [92]. This section discusses the immune-evading properties of biomimetic coatings that provide bPNMs the capacity for such pronounced tumor selectivity.

**Figure 5:**
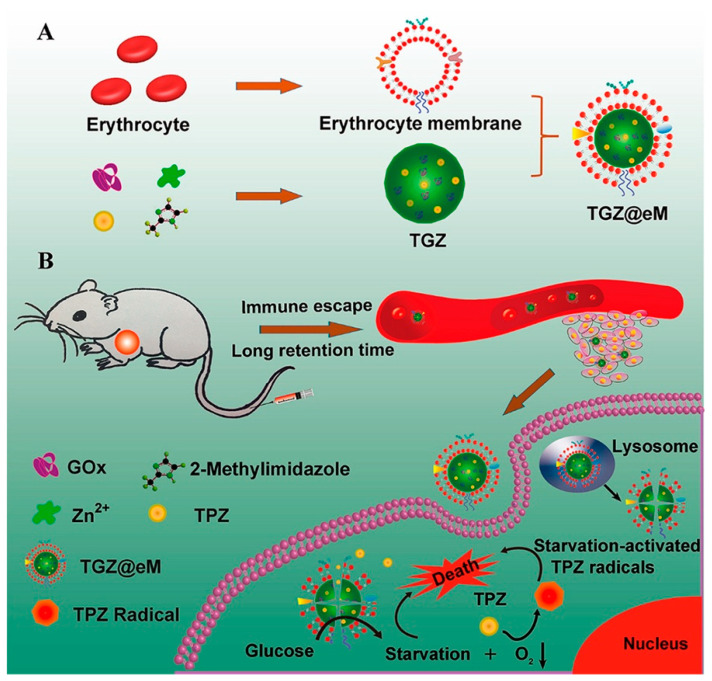
Illustration of immune evasion by RBC membrane camouflaged TGZ (**A**) synthesis of TGZ@eM nanoreactor and (**B**) immune escape by TGZ@eM nanoreactor when administered intravenously to the tumor region. Reproduced with permission from [14], ACS nano, 2018.

RBC coated membranes are known to express CD47, a marker which sends a “don’t eat me” signal to macrophages during the systemic circulation through an interaction with signal regulatory protein-alpha (SIRP-a) receptors. In a study by Wang et al., membrane CD47 was also reported as a major contributor to evasion of macrophage uptake and enhanced circulation times in vivo [93]. In a study by Zhang et al., the RAW264.7 murine macrophage cell uptake of rhodamine B loaded glucose oxidase and zeolitic imidazolate framework-8 (Rhm B-GOx-ZIF) was about 2.9 times higher than that of an RBC membrane-coated equivalent [14]. In the same study, RBC membrane coatings allowed the nanoconstruct to escape systemic clearance and elevated its tumor uptake by 2 times, as compared to the uncoated construct (Figure 5) [14]. In a study by Sun et al., CD47 was also identified as the membrane protein responsible for prevention of the biomimetic construct from macrophage uptake [12]. In this study, the authors successfully characterized CD47 on the membrane of 4T1 cells using SDS-PAGE and Western blotting.

Wang et al. also reported CD47 as the surface protein on RBC membrane coated photothermal polypyrrole nanoparticles, which enhanced immune evasion, thus resulting in prolonged blood circulation times [94]. A cell uptake assay was carried out using RAW264.7 murine macrophage cells, which showed about 2-fold and 1.5-fold lower uptake of the biomimetic construct, as compared to uncoated constructs for a 0.5 h and 1 h incubation, respectively. The in vivo pharmacokinetic study also showed that the biomimetic constructs exhibited a 10-fold higher uptake as compared to uncoated constructs and PEGylated constructs. The PEGylated constructs had a 4% ID/g blood retention, while the biomimetic constructs had a 10% ID/g blood retention 24 h following intravenous injection. As such, CD47 appears to be a critical determinant in immune evasion, which directly prolongs blood circulation of nanoconstructs and promotes their tumor-selective delivery. In fact, isolated CD47 has even been shown to reduce macrophage uptake of synthetic nanoparticles following surface conjugation [95].

### 3.3. Tumor Tissue Permeating Behavior

**Figure 6:**
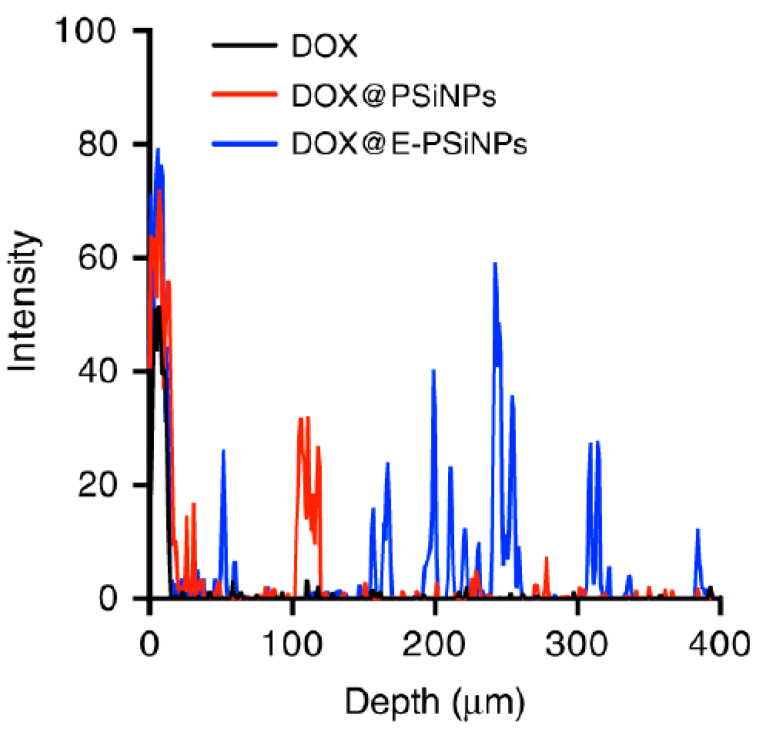
Penetration profile of doxorubicin, uncoated porous silica nanoparticle and exosome coated porous silica nanoparticles. Reprinted from [29], Nature Communications, 2019.

Limited nanoconstruct penetration into the bulk of a tumor mass remains to be one of the major issues in nanomedicines for drug delivery. At the tumor site, tumor vascular network disorganization, the dense ECM barrier and the extensive distribution of stromal cells are the major obstacles that hamper the transport of nanomedicines within the tumor [92]. In a study by Yong et al., the authors developed tumor-cell exosome coated biomimetic porous silica nanoparticles loaded with doxorubicin [29]. The biomimetic construct demonstrated enhanced B16-F10 tumor accumulation and 3–4 fold improved tumor penetration into the parenchyma from blood vessels, as compared to uncoated constructs (Figure 6). This enhanced tumor penetration was attributed in this study to CD54 expressed on exosomes.

Jia et al. reported a highly penetrative biomimetic C6 cell coated liposome nanomedicine M-Lipo-DOX, which was observed to penetrate 2.25-fold deeper than that of doxorubicin-loaded liposomes in a 3D tumor spheroid model of C6 cells [96]. Similarly, numerous other biomimetic constructs have shown enhanced tumor tissue penetration as compared to their uncoated counterparts. These include biomimetic lipoprotein-loading targeting peptide-conjugated cytotoxic mertansine (RM) measured in 4T1-induced tumor mouse models, RBC coated paclitaxel in metastatic breast tumors, RBC coated chemotherapy with iRGD peptide targeting in breast tumors, exosome coated PH20 hyaluronidase in prostate cancer and many others [23,74,97,98]. This attribute of biomimetic constructs is of the most promising for tumor delivery of bPNMs, as tumor tissue penetration remains to be a major hurdle in nanomedicines. Further studies into mechanisms of tumor tissue penetration, as regulated by CD54 or otherwise, will prove to be pivotal for understanding how and why constructs such as bPNMs can capitalize on this unique facet of biomimetic nanotechnology.

## 4. Addressing and Targeting Heterogeneity

As previously mentioned, heterogeneity in the expression of cancer cell surface molecules, coupled to high mutation rates within an individual tumor and between cancer types, can nullify the benefits of homotypic targeting by cancer cell membrane and exosome-based biomimetic constructs. Such cancer cell derived biomimetic constructs ultimately become constrained by their specificity for the parent cancer cell from which they were derived. Therefore, the ‘landscape’ of transmembrane protein concentrations of cancer cells and their respective biomimetic constructs, also referred to as the surfaceome, may in some cancer types primarily be externally distinguishable from healthy tissue by differing expression levels and relative concentrations of the same surface molecules.

In order to successfully translate pre-clinical discoveries in bPNMs, amongst other biomimetic nanotechnologies, it is imperative that a reproducible and efficient methodology is developed for capturing patient cells and for ex vivo expansion and nanoengineering. This is especially important for cancer cell derived biomimetic nanotechnologies whereby homotypic recognition for the patient’s own tumor is central to their value as tumor-specific therapeutics. To some extent, established methodologies for patient-specific CAR-T cell therapy may prove to be invaluable for the adoption of patient-specific biomimetic nanotechnologies. The physical capture and isolation of a cancer cell is also a capture of its surfaceome and portions of the transmembrane receptor clustering cascades associated with it. Since a variety of diagnostic techniques used in the clinic require the capture of whole cancer cells, cell membranes from biopsied tumor tissue may in tandem be repurposed and fractionated into non-replicating, biomimetic nanoconstructs, including bPNMs [99]. Cancers where biopsy is a common diagnostic method include glioblastoma, pancreatic and head and neck cancers [100,101,102]. The biopsy technique most frequently used for glioblastoma diagnosis is the stereotactic needle biopsy, and captured tissue is used as a diagnostic agent via histological analysis [102]. Likewise, cases of pancreatic cancer typically utilize ultrasound- or CT-image guided percutaneous needle biopsies, or endoscopic biopsies, that use isolated cells to histologically diagnose and assess the stage of the disease. Lastly, cases of head and neck cancers most frequently involve fine needle aspiration biopsies, which also use captured cells to diagnose the disease through cytologic examination [100]. Similarly, biomimetic constructs can be derived in a patient-specific manner from cancer cells isolated from routine biopsy procedures. Other clinically relevant methods of cancer cell sampling include the isolation of circulating tumor cells from whole blood, and are also typically used for diagnostic purposes but are limited in availability [103,104]. However, to what extent circulating tumor cells may represent tumor cells in the primary tumor and the heterogeneity within is still under investigation.

Homotypic recognition of cancer cells by biomimetic constructs utilizes the molecular and steric complementarity of multiple cell adhesion molecules simultaneously. We have previously shown that this approach for simultaneous multi-specificity of molecular targeted PNMs is a powerful approach for overcoming receptor heterogeneity in heterotypic cancer nodules. However, given the complexity of both molecular and steric complementarity, homotypic targeting likely increases the chance of tumor cell subpopulations escaping the constraints of the molecular and steric complementarity required for tumor cell specificity. As such, it is imperative that the cancer cells sampled from a patient tumor are truly representative of the surfaceome heterogeneity within tumor cell subpopulations in order for a complete tumor eradication by biomimetic constructs. Importantly, the capture of tumor tissue in a clinical setting also allows for the assessment of molecular heterogeneity among and between cancer cells by methods such as chromogenic immunohistochemistry and quantitative immunofluorescence. Added to that, surface densities of the respective cell adhesion molecules that provides the foundation of the steric complementarity also need to be evaluated and quantified in order to successfully target heterogeneous tumors. Some of the techniques for isolating cancer cells in a clinical setting, such as needle biopsies, are not unlike those used for extracting naive T cells from the blood via leukapheresis during a blood draw for CAR-T cell therapy, which are ultimately reinjected into patients following lymphapheresis [105]. Since small numbers of oncogenic genes are capable of influencing disproportionately large numbers of surface proteins, and several thousand transmembrane proteins have been identified, biomimetic nanoconstructs that more closely mimic the cancer cell surfaceome instead of relying on a single tumor-specific antigen may be at an advantage [106,107,108]. Therefore, as cancer cell sampling procedures improve in parallel with surfaceome sampling and spatial mapping techniques, we envisage that the use of biomimetic nanotechnology, along with NIR PDT-based regimens using bPNMs, will inevitably lead to their clinical adoption.

However, this is not without its challenges. Engineering challenges in the ex vivo scale-up of cancer cell isolates as well as the ex vivo expansion of tumor-specific exosomes for quantities sufficient for therapeutic bPNM preparation will undoubtedly remain as a major limitation to their translation. Furthermore, if engineering processes for the accurate capture and recapitulation of patient tumor heterogeneity were successfully developed, and if such processes enabled sufficient scale-up, limitations due to genetic and phenotypic drift ex vivo will still remain. Owing to the genetic instability of cancer cells and the criticality of the in vivo mechanical and biochemical cues they receive in vivo in order to maintain their surfaceome, the ex vivo expansion of cancer cells and isolation of respective exosomes or membranes for bPNM coatings will prove challenging. Given the delicate surfaceome balance required for homotypic complementarity, even moderate genotypic and phenotypic drifts in the surfaceome of exosomes and cancer cell membranes can prove to annihilate homotypic recognition. Although significantly more complicated, efforts to synthetically re-engineer the surfaceome composition that will ultimately allow for bPNM homotypic recognition in the patient may in fact prove to be a far more robust and reliable approach.

## 5. Perspectives

Given the persistent limitations of synthetic PNMs, amongst other synthetic nanomedicines, as effective drug delivery systems, bPNMs are emerging as a forward-looking approach. bPNMs possess the unique ability to circumvent the limitations of synthetic PNMs through biomimicry of critical biological processes. Salient features of bPNMs that result from their unique biomimicry include (1) unparalleled immune evasion and prolonged circulation half-lives, (2) augmented delivery to solid tumors, (3) active extravasation into tumor tissue, (4) efficient and more homogenous permeation throughout tumor tissue and (5) molecular specificity through homotypic recognition. Taken together, bPNMs have the capacity to outperform any synthetic PNMs developed to date with regards to tumor-selective and tumor-specific delivery. In doing so, bPNMs effectively bypass the substantial bottlenecks that have plagued synthetic nanomedicines throughout the past three decades.

Although they hold substantial promise as tumor-selective and tumor-specific entities for synergistic PDT-based combination regimens, no clear path exists to date for their clinical adoption. By using approaches that have been approved for patient customized cellular therapies, such as patient-specific CAR-T cell therapy, bPNMs may find an expedited pathway to clinical adoption. This strategy may prove to be successful for tumor-selective delivery using erythrocyte membranes and stem cell membranes. However, a major attractive facet of cancer cell membrane-derived bPNMs is their capacity for homotypic recognition and cancer cell specificity in vivo at the cellular level. This specificity is only representative of the cancer cell type from which the bPNMs were formulated from. As such, tumor heterogeneity, especially with regards to the diversity, density and steric complementarity of cell surface adhesion molecules (e.g., TF antigen, Cadherins, galectin-3, EpCAM, Na^+^/K^+^-ATPase, and glycoprotein 100, etc.) may prove to be extremely difficult to address in the clinic. Comprehensively capturing such tumor heterogeneity and accurately recapitulating it within bPNMs, which are capable of homotypic recognition of diverse tumor cell subpopulations, will undoubtedly prove to be the most challenging hurdle in the path to clinical translation. While we have shown that heterogeneity targeting by synthetic, triple receptor-specific PNMs is achievable in vitro, preparing bPNMs with that degree of control over specificity for diverse heterogenous receptors will prove to be problematic in the clinic [109]. It may, however, be the case that the future of bPNMs which are capable of both tumor-selective delivery and tumor-specific homotypic recognition is not in the use of natural cancer cell membrane coatings. Rather, their future may lie in the use of advanced synthetic coatings that are accurately fabricated with the appropriate recombinant cell surface adhesion molecules, at the right density, diversity and steric complementarity that matches the heterogeneity of a particular patient’s tumor. This will undoubtedly be contingent on the accuracy of patient tumor sampling and advanced methods of rapidly screening patient tumors for the presence, density and steric complementarity of cell surface adhesion molecules that are known to facilitate homotypic recognition. That being said, with the appropriate technologies available and with the standardization of synthetic protocols for patient-specific biomimetic nanoconstruct coatings, bPNMs have the potential for presenting themselves as a true next-generation of tumor-selective and tumor-specific NIR PDT-based combination therapies. As biomimetic nanotechnology is further refined at all levels of development, it becomes increasingly likely that upcoming clinical approvals of significantly more efficacious and safer PDT-based combination therapies will comprise NIR activable bPNMs.

## Data Availability

Not applicable.
